# Changes in malaria burden and transmission in sentinel sites after the roll-out of long-lasting insecticidal nets in Papua New Guinea

**DOI:** 10.1186/s13071-016-1635-x

**Published:** 2016-06-14

**Authors:** Manuel W. Hetzel, Lisa J. Reimer, Gibson Gideon, Gussy Koimbu, Céline Barnadas, Leo Makita, Peter M. Siba, Ivo Mueller

**Affiliations:** Papua New Guinea Institute of Medical Research, Goroka and Madang, Papua New Guinea; Swiss Tropical and Public Health Institute, Basel, Switzerland; University of Basel, Basel, Switzerland; Case Western Reserve University, Cleveland, OH USA; Present address: Liverpool School of Tropical Medicine, Liverpool, UK; National Department of Health, Waigani, Papua New Guinea; Population Health and Immunity Division, Walter and Eliza Hall Institute of Medical Research, Parkville, Vic Australia; Department of Medical Biology, University of Melbourne, Parkville, Vic Australia; Present address: European Public Health Microbiology (EUPHEM) training programme, European Centre for Disease Prevention and Control (ECDC), Stockholm, Sweden; Present address: Statens Serum Institut, Copenhagen, Denmark; Barcelona Centre for International Health Research (CRESIB, Hospital Clínic-Universitat de Barcelona), Barcelona, Spain

**Keywords:** Malaria, Insecticide treated nets, *Anopheles*, Papua New Guinea, *Anopheles punctulatus*

## Abstract

**Background:**

Papua New Guinea exhibits a complex malaria epidemiology due to diversity in malaria parasites, mosquito vectors, human hosts, and their natural environment. Heterogeneities in transmission and burden of malaria at various scales are likely to affect the success of malaria control interventions, and vice-versa. This manuscript assesses changes in malaria prevalence, incidence and transmission in sentinel sites following the first national distribution of long-lasting insecticidal nets (LLINs).

**Methods:**

Before and after the distribution of LLINs, data collection in six purposively selected sentinel sites included clinical surveillance in the local health facility, household surveys and entomological surveys. Not all activities were carried out in all sites. Mosquitoes were collected by human landing catches. Diagnosis of malaria infection in humans was done by rapid diagnostic test, light microscopy and PCR for species confirmation.

**Results:**

Following the roll-out of LLINs, the average monthly malaria incidence rate dropped from 13/1,000 population to 2/1,000 (incidence rate ratio = 0.12; 95 % CI: 0.09–0.17; *P* < 0.001). The average population prevalence of malaria decreased from 15.7 % pre-LLIN to 4.8 % post-LLIN (adjusted odds ratio = 0.26; 95 % CI: 0.20–0.33; *P* < 0.001). In general, reductions in incidence and prevalence were more pronounced in infections with *P. falciparum* than with *P. vivax*. Additional morbidity indicators (anaemia, splenomegaly, self-reported fever) showed a decreasing trend in most sites. Mean *Anopheles* man biting rates decreased from 83 bites/person/night pre-LLIN to 31 post-LLIN (*P* = 0.008). *Anopheles* species composition differed between sites but everywhere diversity was lower post-LLIN. In two sites, post-LLIN *P. vivax* infections in anophelines had decreased but *P. falciparum* infections had increased despite the opposite observation in humans.

**Conclusions:**

LLIN distribution had distinct effects on *P. falciparum* and *P. vivax*. Higher resilience of *P. vivax* may be attributed to relapses from hypnozoites and other biological characteristics favouring the transmission of *P. vivax.* The effect on vector species composition varied by location which is likely to impact on the effectiveness of LLINs. In-depth and longer-term epidemiological and entomological investigations are required to understand when and where residual transmission occurs and whether observed changes are sustained.

**Electronic supplementary material:**

The online version of this article (doi:10.1186/s13071-016-1635-x) contains supplementary material, which is available to authorized users.

## Background

Papua New Guinea (PNG) exhibits a diverse and complex malaria epidemiology [1]. Four *Plasmodium* species (*P. falciparum*, *P. vivax*, *P. ovale* and *P. malariae*) are endemic and more than ten *Anopheles* species filling different ecological niches have been incriminated in the parasite’s transmission [2]. Historical malaria control measures in the 1960s and 1970s included indoor residual spraying of insecticides, mass drug administration as well as environmental management in certain areas resulting in substantial initial reductions in malaria in many locations [3, 4]. The cessation of the spraying program in the 1980s coinciding with the decentralization of responsibilities for malaria control as well as emerging resistance of the malaria parasites to commonly used drugs [5, 6] led to a subsequent resurgence, particularly of *P. falciparum* malaria, across most parts of PNG [7].

In 2004, the Government of Papua New Guinea re-intensified its malaria control efforts with the financial support of a round 3 grant from the Global Fund to Fight AIDS, Tuberculosis and Malaria. The central component of this program was the first country-wide free distribution of long-lasting insecticidal mosquito nets (LLIN) [8]. Insecticide-treated nets had been shown to reduce the incidence and prevalence of falciparum-malaria in children in PNG as early as 1985 [9]. Yet, while these results were later reflected in policy documents [10], little effort was made in practice to scale up mosquito net use. Coverage with mosquito nets, particularly insecticide-treated nets, therefore remained patchy and low in most parts of the country until 2004 [11–13]. The Global Fund supported program that subsequently facilitated a single round of LLIN distribution resulted in an increase in ownership and use of bednets, particularly LLINs. A national household survey after the distribution found 80 % of all households owning a mosquito net of any type and 65 % owning a LLIN, while 33 % of people reported to sleep under a LLIN [8]. In six sentinel surveillance sites established by the Papua New Guinea Institute of Medical Research (PNGIMR), average LLIN ownership increased from 9 % before the distribution to 89 % thereafter and LLIN use rose from 6 to 55 % [8].

This manuscript assesses malaria morbidity and transmission indicators in sentinel sites before and after the LLIN distribution. The study was part of the evaluation of the Global Fund round 3 malaria grant.

## Methods

### Study sites

**Fig. 1:**
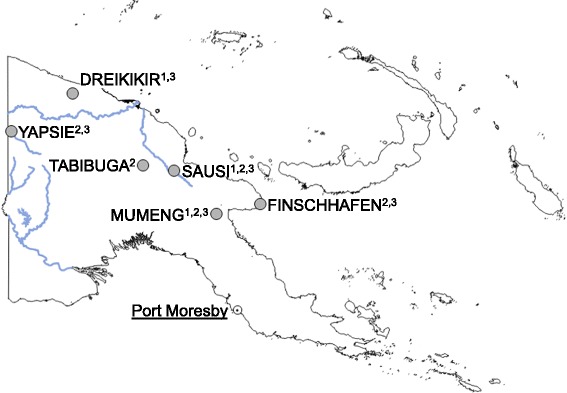
Location of sentinel sites used for (1) clinical surveillance, (2) household surveys, and (3) entomological surveys. Major rivers and lake in *blue*

Sentinel site locations were selected purposively in 2008 in places which were yet to be covered with the LLIN campaign. The sites were located in the Momase and Highlands regions of the main island of PNG (Fig. 1) and consisted of three to four randomly selected villages in the catchment area of a sentinel health facility. Five sites were located in Momase: Dreikikir in the hills on the edges of the Sepik River basin (altitude 200–400 m), Finschhafen on the Morobe coastline of the Huon peninsula (altitude 0–50 m), Sausi in the Ramu River valley (altitude 100–250 m), Mumeng at an intermediate altitude (450–1,550 m) along the road to Bulolo and Wau and Yapsie/Yapsiei near the Indonesian border along a tributary of the Sepik River (altitude 150–250 m). Tabibuga in the Jimi Valley was the only site in the Highlands (altitude 1,350–1,500 m).

### Data collection

Data were collected prior to the LLIN campaign (10/2008–08/2009) and one year later after LLINs had been distributed (10/2009–08/2010). Data collection included clinical surveillance in the local health centre in three sites (Dreikikir, Mumeng and Sausi) over a period of approximately two months pre- and post-LLIN, coinciding roughly with the main malaria transmission season. A household survey in two to three randomly selected villages in the health centre’s catchment area was carried out in five sites (Finschhafen, Mumeng, Sausi, Tabibuga and Yapsie) and entomological surveys were conducted in five sites (Dreikikir, Finschhafen, Mumeng, Sausi and Yapsie). For operational reasons not all activities could be carried out in all sites (Additional file 1: Table S1).

### Clinical surveillance

For the clinical surveillance, all patients attending the health facility were screened for a history of fever within the previous three days. A research nurse collected a finger-prick blood sample from all consenting fever patients and malaria was diagnosed by rapid diagnostic test (RDT; ICT Malaria Combo, ICT Diagnostics, South Africa). From the same blood sample, thick and thin blood films were prepared on one slide and haemoglobin (Hb) levels were measured using a portable analyser (HemoCue Hb 201+, HemoCue AB, Sweden). Anaemia and severe anaemia were defined in accordance with World Health Organization definitions applying age group-specific cut-offs and altitude adjustment [14]. A dry blood spot was prepared on Whatman 3MM filter paper (GE Healthcare), whenever possible, and stored in individual plastic zip-bags with desiccant silica gel. Axillary temperature was measured with an electronic thermometer, and in patients between 2 and 9 years of age the spleen was palpated and graded according to Hackett [15]. Demographic details of the patient were recorded in a paper form. The subsequent clinical assessment, final diagnosis and treatment were then taken over by the health facility’s clinician following standard procedures and results were recorded on the patient’s study form.

### Household survey

For the household survey, three (in the case of Yapsie four) villages were randomly selected from the sentinel health facility’s catchment area. In each village, 30 to 35 households were randomly sampled from a list of all households. A structured questionnaire about the coverage with malaria control interventions was administered to the heads of sampled households. A finger-prick blood sample was collected from household members above five months of age for preparing a microscopy slide, a dry blood spot on filter paper, and for measuring Hb levels as described above. Symptomatic individuals were diagnosed on the spot using a malaria RDT and positive cases were treated according to standard treatment guidelines [16]. Axillary temperature was measured with an electronic thermometer. Details of the household survey methodology have been described in more details elsewhere [8, 17].

### Entomological surveys

Entomological surveys were conducted in two villages per site. Mosquitoes were collected by outdoor human landing catch from 6 households per village prior to the LLIN distribution and again 12 months later. The number of person-nights per collection ranged from 16 to 48. Collectors worked in pairs with one member collecting from 18:00 to 24:00 h and the second collecting from 24:00 to 06:00 h. Mosquitoes were stored per collection hour and identified to morphological species before storage on silica gel. Lysates from whole mosquitoes were screened for *P. falciparum*, *P. vivax* 210 and *P. vivax* 247 circumsporozoite proteins by enzyme-linked immunosorbent assay [18]. DNA was extracted from a portion of the lysate using DNEasy blood and tissue kit (QIAGEN, Maryland, USA). Mosquitoes that were morphologically identified as members of the *An. punctulatus* group were confirmed to species by polymerase chain reaction (PCR) restriction length polymorphism of the ITS2 region [2].

### Laboratory procedures

Thin blood smears were first fixed with methanol and thick and thin smears stained with Giemsa and read by light microscopy independently by two PNGIMR microscopists. Discordant reads were confirmed by a senior microscopist. The number of parasites was counted until reaching 200 white blood cells and a slide was declared negative only after reading a minimum of 200 thick film fields. Molecular diagnosis was used to complement missing second reads and to disambiguate discordant species read results (clinical surveillance *n* = 127; household survey *n* = 87). DNA was extracted from filter papers using the DNeasy Blood and Tissue Kit (Qiagen, Valencia, USA) or the Favorgen 96-Well Genomic DNA Kit (Favorgen Biotech Corp., Taiwan) following the manufacturer’s protocols. The molecular assay was a semi-quantitative post-PCR, ligase detection reaction/fluorescent microsphere assay described in more detail elsewhere [19–21].

### Data analysis

Data were double-entered into a Microsoft FoxPro or Microsoft Access database at PNGIMR Goroka and analysed with Stata (StataCorp, USA) software. Differences in binary and categorical variables between study sites were assessed by Chi-square test and logistic regression, differences in continuous variables by *t*-test and linear regression and a non-parametric test for median age. Multivariable logistic and linear regression models assessing changes in malaria prevalence, parasite density and morbidity indicators were adjusted for age group and study site, anaemia furthermore for sex. In the clinical surveillance samples, primary diagnosis of malaria was based on the result of the RDT as performed in routine clinical practice while species-results were based on light microscopic/PCR diagnosis, which comprised only a sub-set of patients in the case of Mumeng post-LLIN. For the calculation of incidence rates, a stable population denominator was used for both years in the absence of available village-level growth rates.

## Results

### Study sample

Clinical surveillance in three sites included 1,325 fever patients pre-LLIN and 680 post-LLIN. The household surveys in five sites included blood samples from 1,967 individuals pre-LLIN and 1,986 individuals post-LLIN. Pre-LLIN and post-LLIN samples had comparable age distributions in all sites with the exception of the Mumeng clinical surveillance sample (*P* = 0.02). Sample details including LLIN coverage by site are provided in Additional file 2: Table S2. Entomological surveys collected 15,481 anophelines pre-LLIN and 6,066 anophelines post-LLIN. Due to the high density of mosquitoes collected in Sausi, only 34 % (*n* = 5,036) of the pre-LLIN collection in this site and 43 % (*n* = 2,558) of the post-LLIN collection were screened for *Plasmodium* spp. infection.

### Malaria incidence and morbidity in health facilities

Across the three sentinel health facilities (Dreikikir, Mumeng, Sausi), the average proportion of fever patients with a positive RDT decreased from 57.3 % (95 % CI: 54.6–60.0) pre-LLIN to 17.9 % (95 % CI: 15.1–21.0) post-LLIN resulting in a 87 % drop in the pooled crude monthly incidence rate from 13/1,000 population to 2/1,000 (incidence rate ratio IRR = 0.12; 95 % CI: 0.09–0.17; *P* < 0.001) (Table 1).

**Table 1:** Crude monthly malaria incidence rate (IR) in three sentinel health facilities. Diagnosis by RDT

Site	Patients	Pre-LLIN			Post-LLIN			Change	
		*n*	RDT+/ Month	IR/1,000	*n*	RDT+/ Month	IR/1,000	/1,000	%
Dreikikir	8,300	377	178	21.5	290	27	3.3	-18.2	-84.7
Mumeng	17,000	469	102	6.0	215	11	0.6	-5.4	-90.0
Sausi	6,700	458	122	18.2	172	12	1.8	-16.4	-90.1
Overall	32,000	1,304	402	12.6	677	50	1.6	-11.0	-87.3

Based on PCR-corrected light microscopy, the reduction was more pronounced in the proportion of fever cases infected with *P. falciparum* (46.8 to 9.1 %; adjusted odds ratio AOR = 0.10; 95 % CI: 0.07–0.14; *P* < 0.001) than with *P. vivax* (12.7 to 6.9 %; AOR = 0.59; 95 % CI: 0.40–0.85; *P* < 0.001). Reductions in *P. vivax* infections were statistically significant only in children below 5 years of age in two sites (Mumeng AOR = 0.09, 95 % CI: 0.01–0.64; *P* = 0.017; Sausi AOR = 0.36; 95 % CI: 0.15–0.91) but not in older age groups (all sites AOR = 0.62; 95 % CI: 0.34–1.14; *P* = 0.122). In Dreikikir, there was no significant reduction in *P. vivax* in any age group (AOR = 0.87; 95 % CI: 0.51–1.48; *P* = 0.6). The decrease in infections with *P. malariae* was substantial but due to low numbers of cases not statistically significant (2.1 to 0.3 %; AOR = 0.24; 95 % CI: 0.06–1.04; *P* = 0.057).

**Fig. 2:**
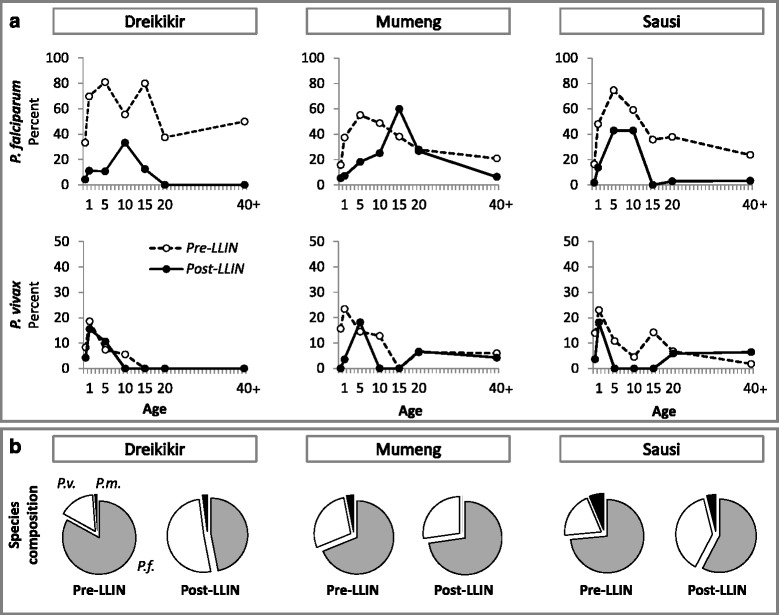
**a** Prevalence of malaria in fever patients by site and age group before (*dotted line*) and after (*solid line*) LLIN distribution. **b** Species distribution by site. *Abbreviations*: *P.f.*, *P. falciparum* (*grey*), *P.v.*, *P. vivax* (*white*), *P.m.*, *P. malariae* (*black*)

The parasite species composition in the febrile patient sample changed in Dreikikir (but not in the other sites) from a clear dominance of *P. falciparum* over *P. vivax* (67.9 *vs* 12.9 %, Fisher's exact test, *P* < 0.001) to equal proportions of the two species (7.9 *vs* 8.6 %, *P* = 0.88). Pre-LLIN, *P. falciparum* infection prevalence peaked in fever patients aged 5–9 years, and *P. vivax* in 1–4 year olds. Post-LLIN, the *P. falciparum* peak had shifted to later age groups, while for *P. vivax* infections a clear peak could not be identified due to the low number of positive patients. Changes in test-positivity and species composition by site are shown in Fig. 2.

**Fig. 3:**
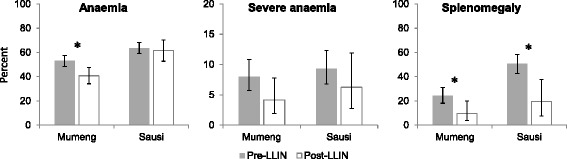
Indicators of morbidity in fever patients before and after LLIN distribution. Error bars show 95 % confidence intervals. *P < 0.05

In two sites (Mumeng and Sausi), anaemia and splenomegaly were assessed in the fever patients. A statistically significant reduction was found in anaemia in Mumeng (52.9 to 40.7 %; AOR = 0.6; 95 % CI: 0.5–0.9; *P* = 0.015), and in splenomegaly (all grades) in both sites (pooled 36.9 to 12.9 %; AOR = 0.3; 95 % CI: 0.1–0.5; *P* < 0.001). Reductions in anaemia in Sausi and of severe anaemia in both sites were not statistically significant (Fig. 3). Details by site are provided as supplementary material (Additional file 3: Table S3).

### Malaria prevalence and morbidity in the general population

**Fig. 4:**
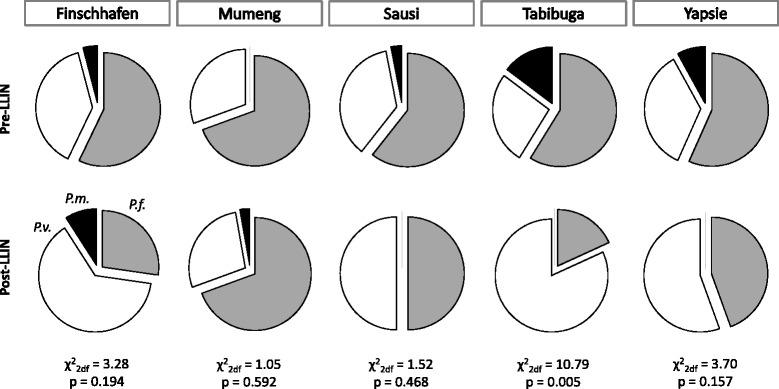
*Plasmodium* species composition in infected community members before (top) and after (bottom) LLIN distribution. *Abbreviations*: *P.f.*, *P. falciparum* (*grey*), *P.v.*, *P. vivax* (*white*), *P.m.*, *P. malariae* (*black*)

Across five sentinel sites, the average prevalence of malaria by light microscopy in the general population of the health facility catchment areas decreased from 15.7 % pre-LLIN to 4.8 % post-LLIN (AOR = 0.26; 95 % CI: 0.20–0.33; *P* < 0.001). The largest reduction was observed in Yapsie, the site with the highest pre-LLIN prevalence of both *P. falciparum* (18.9 %) and *P. vivax* (11.8 %). Across all sites, the reductions were more pronounced in infections with *P. falciparum* than with *P. vivax* (Table 2). The changes in *P. vivax* infections were only statistically significant in two sites (Finschhafen and Yapsie) (Table 2). In three sites (Finschhafen, Tabibuga and Yapsie), there was a change from *P. falciparum* dominance to *P. vivax* dominance, which reached statistical significance (*P* = 0.005) only in Tabibuga (Fig. 4). There was no statistically significant difference in the observed reductions between age groups or sex (Mantel Haenszel test of homogeneity of odds ratios, all *P* > 0.1).

**Table 2:** Malaria prevalence in the general population before and after LLIN distribution

Site	Pre-LLIN		Post-LLIN			
	*n*	% (95 % CI)	*n*	% (95 % CI)	AOR	*P*-value
Any species						
Finschhafen	455	9.9 (7.3–13.0)	442	2.5 (1.2–4.4)	0.16 (0.07–0.34)	< 0.001
Mumeng	290	10.0 (6.8–14.0)	462	7.8 (5.5–10.6)	0.62 (0.36–1.07)	0.086
Sausi	337	9.2 (6.3–12.8)	422	4.7 (2.9–7.2)	0.48 (0.26–0.88)	0.017
Tabibuga	325	9.8 (6.8–13.6)	341	3.2 (1.6–5.7)	0.25 (0.12–0.52)	< 0.001
Yapsie	560	30.5 (26.7–34.5)	319	5.6 (3.4–8.8)	0.10 (0.06–0.18)	< 0.001
Overall	1,967	15.7 (14.1–17.3)	1,986	4.8 (3.9–5.9)	0.26 (0.20–0.33)	< 0.001
*P. falciparum*						
Finschhafen	455	6.2 (4.1–8.8)	442	0.7 (0.1–2.0)	0.10 (0.03–0.33)	< 0.001
Mumeng	290	8.6 (5.7–12.5)	462	5.4 (3.5–7.9)	0.49 (0.27–0.91)	0.023
Sausi	337	5.9 (3.7–9.0)	422	2.6 (1.3–4.6)	0.46 (0.21–0.98)	0.044
Tabibuga	325	6.2 (3.8–9.3)	341	0.6 (0.1–2.1)	0.08 (0.02–0.34)	0.001
Yapsie	560	18.9 (15.8–22.4)	319	2.5 (1.1–4.9)	0.12 (0.05–0.24)	< 0.001
Overall	1,967	10.1 (8.8–11.5)	1,986	2.5 (1.8–3.2)	0.23 (0.16–0.32)	< 0.001
*P. vivax*						
Finschhafen	455	4.2 (2.5–6.4)	442	1.6 (0.6–3.2)	0.25 (0.09–0.68)	0.007
Mumeng	290	3.8 (1.9–6.7)	462	2.2 (1.0–3.9)	0.44 (0.18–1.07)	0.071
Sausi	337	3.6 (1.9–6.1)	422	2.6 (1.3–4.6)	0.67 (0.28–1.58)	0.357
Tabibuga	325	2.8 (1.3–5.2)	341	2.6 (1.2–5.0)	0.84 (0.32–2.20)	0.728
Yapsie	560	11.8 (9.2–14.7)	319	3.1 (1.5–5.7)	0.18 (0.08–0.39)	< 0.001
Overall	1,967	5.9 (4.9–7.1)	1,986	2.4 (1.7–3.1)	0.38 (0.26–0.55)	< 0.001
*P. malariae*						
Finschhafen	455	0.4 (0.1–1.6)	442	0.2 (0–1.3)	0.51 (0.05–5.68)	0.587
Mumeng	290	0 (0–1.3)	462	0.2 (0–1.2)	na	
Sausi	337	0.3 (0–1.6)	422	0 (0–0.9)	na	
Tabibuga	325	1.5 (0.5–3.6)	341	0 (0–1.1)	na	
Yapsie	560	2.7 (1.5–4.4)	319	0 (0–1.1)	na	
Overall	1,967	1.2 (0.7–1.7)	1,986	0.1 (0–0.4)	0.09 (0.02–0.36) ^a^	0.001

^a^ unadjusted odds ratio

na, not available

**Fig. 5:**
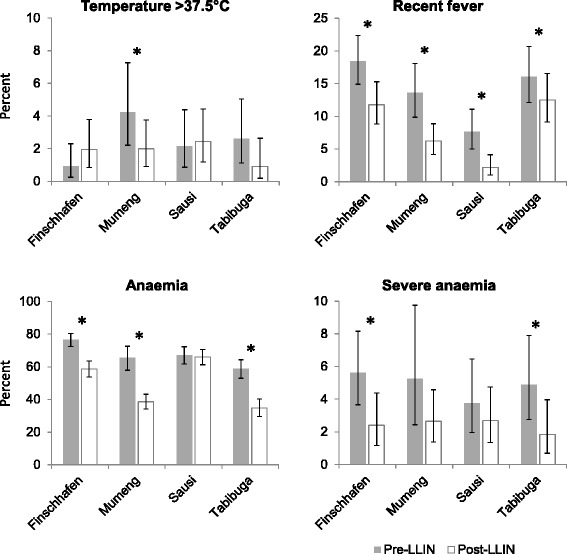
Indicators of morbidity in the general population before and after LLIN distribution. Error bars show 95 % confidence intervals. *P < 0.05

Axillary temperature, self-reported fever and anaemia were assessed as additional morbidity indicators in the general population in four sites (Finschhafen, Mumeng, Sausi, Tabibuga). Acute fever, defined as axillary temperature of > 37.5 °C, was measured in 2.3 % (95 % CI: 1.6–3.2) of individuals pre-LLIN and 1.9 % (95 % CI: 1.3–2.7) post-LLIN (AOR = 0.72; 95 % CI: 0.43–1.20; *P* = 0.208). Of those infected with malaria parasites, 12.9 % (95 % CI: 7.7–19.8) had an acute fever pre-LLIN and 8.1 % (95 % CI: 3.0–16.8) post-LLIN (AOR = 0.30; 95 % CI: 0.09–0.95; *P* = 0.041). Self-reported recent febrile illness (previous 2 days) was reported more frequently, by 14.3 % (95 % CI: 12.5–16.3) pre-LLIN and 7.9 % (95 % CI: 6.6–9.3) post-LLIN (AOR = 0.5; 95 % CI: 0.4–0.7; *P* < 0.001). A reduction was also noted in anaemia from 68.3 % (95 % CI: 65.6–70.9) to 50.1 % (95 % CI: 47.6–52.6) (AOR = 0.4; 95 % CI: 0.4–0.5; *P* < 0.001), and in severe anaemia from 4.9 % (95 % CI: 3.8–6.2) to 2.4 % (95 % CI: 1.7–3.3) (AOR 0.5; 95 % CI: 0.3–0.7; *P* < 0.001). Not all reductions reached statistical significance in all sites, partly due to small sample sizes (Fig. 5). Details by site are provided as supplementary material (Additional file 4: Table S4).

### Malaria transmission

**Fig. 6:**
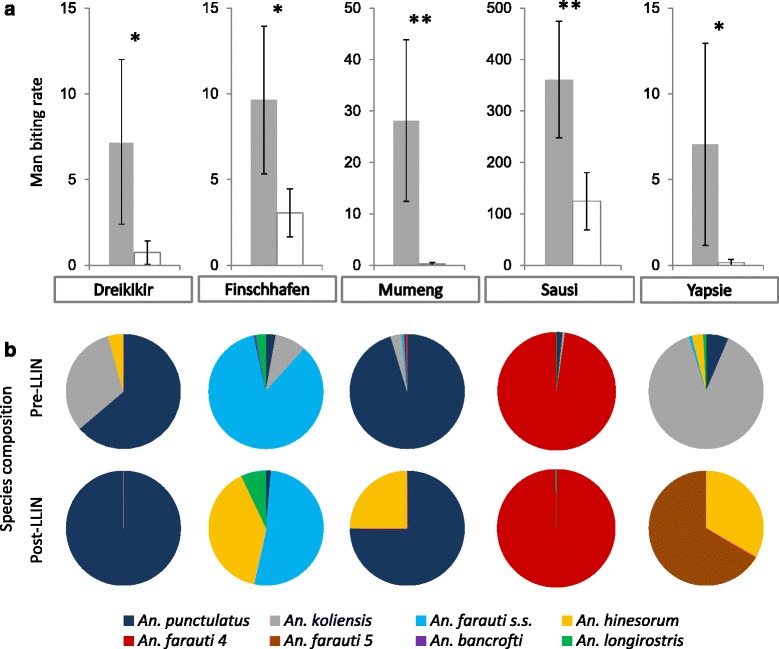
**a** Mean nightly *Anopheles* man biting rate (MBR; bites/person/night) before (*grey*) and after (*white*) LLIN distribution. **b** *Anopheles* species composition before and after LLIN distribution. Error bars show 95 % confidence intervals. *P < 0.05; **P < 0.001

Mean *Anopheles* man biting rates (MBR) across five sites decreased from 83 (95 % CI: 48– 117) bites/person/night pre-LLIN to 31 (95 % CI: 15–47) bites/person/night post-LLIN (*t*_(141)_ = 2.69, *P* = 0.008). The highest mean pre-LLIN MBR (361/person/night) was found in Sausi and dominated by *An. farauti* 4 (Fig. 6). Reductions were greatest in biting rates of *An. koliensis* (none collected post-LLIN), *An. punctulatus* (-93.3 %) and *An. farauti* (*s.s.*) (formerly *An. farauti* 1; -76.2 %), while *An. farauti* 4 (-48.1 %), present in large numbers in Sausi, and *An. longirostris* (-6.3 %) appeared least affected. *An. hinesorum* and *An. farauti* 5 were mainly found post-LLIN. Diversity in species composition was lower after the LLIN distribution and in all sites except Yapsie, the species dominant pre-LLIN remained dominant post-LLIN (Fig. 6).

Prior to LLIN roll-out, the majority of infected mosquitoes in three sites (Finschhafen, Mumeng and Sausi) carried *P. vivax* sporozoites, while in two sites (Dreikikir and Yapsie) *P. falciparum* infections were dominant. Sporozoite prevalence was lowest in Sausi, where the MBR was highest. Post-LLIN, no infected mosquitoes were found in Dreikikir, Mumeng and Yapsie. In the remaining sites, pre-LLIN infection of mosquitoes and monthly entomological inoculation rate (EIR) were higher for *P. vivax* than for *P. falciparum*. Post-LLIN, *P. vivax* infections had decreased, but *P. falciparum* infections had increased in both sites (Table 3).

**Table 3:** Anopheles nightly man biting rate and malaria transmission before and after LLIN distribution

	Pre-LLIN						Post-LLIN						Change	
Site			Prevalence of infection (%)		Monthly EIR				Prevalence of infection (%)		Monthly EIR		MBR	
	*An* (*n*)	*An* MBR	*P.f.*	*P.v.*	*P.f.*	*P.v.*	*An* (*n*)	*An* MBR	*P.f.*	*P.v.*	*P.f.*	*P.v.*		%
Dreikikir	144	7.2	3.4	1.7	7.3	3.7	12	0.8	0	0			-7	-90
Finschhafen	212	9.6	0.5	2.7	1.6	7.9	67	3.0	3.2	0	2.9	0.0	-7	-68
Mumeng	562	28.1	2.2	3.7	18.9	31.5	5	0.3	0	0			-28	-99
Yapsie	127	7.1	2.4	0.8	5.0	1.7	3	0.2	0	0			-7	-98
Sausi	14,436	360.9	0.02	0.2	2.2	23.7	5,979	124.6	0.2	0.04	5.8	1.5	-236	-65

*Abbreviations*: *An Anopheles*, *P.f. Plasmodium falciparum*, *P.v.*, *P vivax*, *MBR* man biting rate (bites/person/night), *EIR* entomological inoculation rate

## Discussion

The roll-out of LLINs in 2009 was followed by substantial reductions in key indicators of malaria morbidity and transmission in six sites in PNG within less than one year. Average LLIN use in these sites had increased from 6 to 55 %, and 89 % of households in the sites owned at least one LLIN after the distribution [8]. The LLINs were new and efficacious [22] and there was no indication of resistance to pyrethroids in PNG, even though data at that time was limited [23]. The prevalence of *Plasmodium* spp. infection in the general population dropped significantly over the same period mainly due to a 76 % reduction in infections with *P. falciparum* (AOR = 0.23, *P* < 0.001). The prevalence of blood-stage *P. vivax* parasitaemia decreased less prominently (-60 %) reaching statistical significance in two of five study sites (Finschhafen and Yapsie). A significant reduction was also observed in the incidence of clinical malaria cases in three sites, primarily as a result of an 81 % drop in the proportion of *P. falciparum*-infected febrile patients (AOR = 0.1, *P* < 0.001). The proportion of cases infected with *P. vivax* decreased less across all age groups (-46 %), and statistically significantly only in children under 5 years of age. In the Dreikikir site, no significant decrease in the proportion of *P. vivax* cases was detected in any age group. These findings confirm results from a cohort study in young children conducted near Dreikikir which found that LLIN use was associated with a reduction in both *P. falciparum* infections and clinical episodes, while it only reduced *P. vivax* infections but not clinical episodes [24].

A relative increase in *P. vivax* over *P. falciparum* following implementation of malaria control measures confirms previous observations both from PNG [7, 9, 20, 25] and elsewhere [26, 27]. The shift in species composition was observed in both asymptomatic community members and febrile patients although not to the same degree in all sites. The differential impact of interventions on the two parasites species is likely a result of the parasite’s [28] and potentially the vector’s biology [29].

The ability of *P. vivax* to relapse from long-lasting liver stages and the higher probability of gametocytaemia are most likely key factors. A recent study confirmed that relapses from *P. vivax* hypnozoites contribute 80 % of the burden of malaria infection and clinical episodes in PNG [30]. In tropical strains, such as the South-West Pacific Chesson strains, relapses occur rapidly and frequently [31]. Although the majority of hypnozoites may therefore activate within 12 months of an initial infection, hypnozoites can survive in the human liver for up to 3–5 years. As a consequence, *P. vivax* blood-stage infections detected post-LLIN roll-out are not only due to new infections from mosquito bites but also due to relapses from hypnozoites established both pre- and post-LLIN [32]. Gametocytaemia was previously found to be driven by asexual blood stage parasitaemia, with a 10-fold increase in parasite density leading to a 1.8-fold and 3.3-fold increase in the odds of carrying *P. falciparum* and *P. vivax* gametocytes, respectively [33]. Reducing the chance of *P. vivax* transmission would therefore require a stronger reduction of *P. vivax* parasitaemia. Also, *P. vivax* gametocytes appear early in an infection, when asexual densities are still very low [34] and almost all *P. vivax* infections are thus thought to be gametocyte positive [35] hence increasing the probability of transmission. Previous studies in PNG further found evidence of earlier biting *Anopheles* mosquitoes being more likely infected with *P. vivax* than *P. falciparum* [29, 36], thus increasing the relative probability of *P. vivax* transmission before people retire to bed. This is further aggravated by the shorter extrinsic incubation period for *P. vivax*. As LLINs reduce the biting density as well as the lifespan of mosquitoes, an equal reduction in lifespan is more likely to interrupt *P. falciparum* transmission [37]. As a consequence of the above, particularly indoor vector control interventions are likely to have less impact on *P. vivax* than *P. falciparum* gametocytaemia and hence on transmission. However, once the pre-LLIN hypnozoite reservoir is exhausted, *P. vivax* prevalence can be expected to drop in line with reductions in the *P. vivax* EIR.

The entomological results indicate a significant impact of LLINs on transmission but a varying effect by species and study site. In the site with the largest number of mosquitoes pre- and post-LLIN (Sausi, *An. farauti* four in both rounds), only the *P. vivax* EIR was found to decrease after LLIN distribution, while the *P. falciparum* EIR increased. The same was observed in Finschhafen, yet with a small number of mosquitoes post-LLIN (*n* = 67). In the other sites the mosquito sample size post-LLIN was too limited to draw strong conclusion. However, more comprehensive entomological data published by Reimer et al. [38] confirmed the parasite species shift in *Anopheles* infections from *P. vivax* pre-LLIN to *P. falciparum* one to two years post-LLIN in Madang province and Dreikikir. The different baseline species composition reported by the two studies for Dreikikir is likely related to the low number of infected mosquitoes (two *P. falciparum*, one *P. vivax*) caught pre-LLIN in this study. At the same time, a comparison of results from remote Yapsie(i) area, where a 1986 survey in three villages had found 0.4–1.5 % of anophelines (mostly *An. koliensis*) to be infected with *P. falciparum* and 0–1.7 % with *P. vivax* [39], confirms the short term impact of LLINs on both species in areas with well-established transmission*.* Interestingly in that site, neither in 1986 nor in 2009 infected were mosquitoes collected in the central Yapsie station.

Further in-depth entomological and epidemiological investigations over longer periods of time are needed to clarify the relationship between infections in mosquitoes and in humans. Sub-microscopic infections in humans may explain part of the differences in parasite species detected in humans and mosquitoes [30, 40]. A better understanding is urgently required of when and where (at both macro- and micro-levels) human-mosquito contacts result in malaria transmission in order to target both vector control and vector surveillance. It remains unclear how well current entomological monitoring practices capture actual transmission in space and time. A longer-term follow-up will be required to measure the full impact of LLINs on *P. vivax* prevalence, incidence, and transmission.

Few additional morbidity indicators were assessed in this study, all of which showed a reduction in frequency (even though not all reached statistical significance) suggesting generally positive health developments. Splenomegaly, a syndrome closely associated with chronic malaria in PNG [41, 42], was less frequent among malaria patients in two sites following LLIN distribution. The trend in anaemia differed between sites. This study confirmed previous findings that anaemia is highly prevalent in some parts of PNG [17, 43, 44] with pre-LLIN prevalence of mild to moderate anaemia between 59 and 77 %. The prevalence of anaemia decreased in the general population (and in fever patients in Mumeng) post-LLIN. No statistically significant decrease was found in Sausi, interestingly the site in which parasite density in malaria patients remained higher than in the two other sites with clinical surveillance. However, the aetiology of anaemia is known to be multi-factorial and a causal link between LLIN and anaemia is likely to be confounded by a number of other contributing factors. Manning et al*.* [44] for example identified parvovirus B19 infection, *P. falciparum* infection, vitamin A deficiency, wasting, and incomplete vaccination as primary risk factors for severe anaemia in PNG. As expected, the vast majority of infections were asymptomatic both pre- and post-LLIN.

This study was not without limitations. Due to financial and operational constraints not all data collection components (clinical surveillance, household survey, entomology) could be implemented in all study sites, thus limiting the extent to which trends in these indicators can be compared. Household survey and entomology survey were cross sectional in nature and the clinical surveillance limited to approximately three months each round. While this approach effectively disregards seasonality, pre-LLIN and post-LLIN assessments were implemented during the same time of the year in an effort to maximize comparability. Differences in weather patterns potentially impacting on entomological indicators could not be reliably investigated due to a lack of detailed weather records (U.S. National Centers for Environmental Information; http://www.ncdc.noaa.gov/), but rainfall data from Madang airport presented by Reimer et al*.* [38] indicates no abnormalities in the seasonal fluctuations over the study period. However, as the comparison of different entomology datasets from Dreikikir shows, small differences in data collection may alter results and care should therefore be taken when making inferences particularly when measurements are based on small numbers of samples, in this specific case number of mosquitoes. The design of this study does not allow establishing a causal link between the LLIN and the observed health effects. However, to the best of our knowledge no other important health interventions were implemented in the study areas over the course of the two data collection rounds making it highly probable that much of the observed reductions in malaria morbidity and transmission is linked to this single intervention.

## Conclusions

The distribution of LLIN had distinct effects on *P. falciparum *and *P. vivax*. Higher resilience of *P. vivax *may be attributed to relapses from hypnozoites and other biological characteristics favouring the transmission of *P. vivax*. The effect on vector species composition varied by location which is likely to impact on the effectiveness of LLINs. The relationship between prevalence, incidence and transmission is a function of several factors, including many that could not be investigated in the frame of this study. The findings of this analysis however clearly show the complexity of these relationships in PNG. Heterogeneity in the impact of LLINs across different sites is obvious from the data presented in this article. Care should therefore be taken when extrapolating findings generated in a particular site in PNG to the entire country. In the past, most in-depth malaria studies in PNG have been conducted in East Sepik (Maprik area) and Madang provinces (Madang North coast area). Gathering data from a larger number of locations over multiple years therefore appears essential to improve our understanding of the malaria epidemiology in PNG particularly as some of the observed effect, such as the shift to *P. vivax* as the most prevalent *Plasmodium* spp. infection, may be transient.

## Abbreviations

AOR, adjusted odds ratio; CI, confidence interval; DNA, Deoxyribonucleic acid; EIR, entomological inoculation rate; Hb, haemoglobin; IR, Incidence rate; IRR, incidence rate ratio; LLIN, long-lasting insecticidal net; MBR, Man biting rate; PCR, Polymerase chain reaction; PNG, Papua New Guinea; PNGIMR, Papua New Guinea Institute of Medical Research; RDT, rapid diagnostic test.

## Additional files

Additional file 1: Table S1. Data collection periods by study site. (DOCX 18 kb) Additional file 2: Table S2. Description of study population by site. (XLSX 15 kb) Additional file 3: Table S3. Morbidity indicators in two sentinel health facilities before and after LLIN distribution. (DOCX 15 kb) Additional file 4: Table S4. Morbidity indicators in the general population before and after LLIN distribution. (DOCX 19 kb)
