# Supplementary material for: Changes in malaria burden and transmission in sentinel sites after the roll-out of long-lasting insecticidal nets in Papua New Guinea
Source: Parasit Vectors. 2016 Jun 14;9:340. doi: 10.1186/s13071-016-1635-x (PMC4908799; doi:10.1186/s13071-016-1635-x)
Supplement: Additional file 1: Table S1. — Data collection periods by study site. (DOCX 18 kb) [file 13071_2016_1635_MOESM1_ESM.docx]

**Additional file 1: Table S1** Data collection periods by study site

| **Province** | **Site** | **Clinical**  **surveillance** | | **Household**  **survey** | | **Entomology**  **survey** | |
| --- | --- | --- | --- | --- | --- | --- | --- |
|  |  | **Pre-**  **LLIN** | **Post-**  **LLIN** | **Pre-**  **LLIN** | **Post-**  **LLIN** | **Pre-**  **LLIN** | **Post-**  **LLIN** |
| East Sepik | Dreikikir | 30.10.–19.12.08 | 23.11.09–17.02.10 |  |  | Nov 08 | Nov 09 |
| Morobe | Finschhafen |  |  | Jun 09 | Jul 10 | Jun 09 | Jun 10 |
| Morobe | Mumeng | 28.01.–31.03.09 | 21.01.–18.03.10 | Feb 09 | Mar 10 | Feb 09 | Mar 10 |
| Madang | Sausi | 20.10.–18.12.08 | 21.10.–17.12.09 | Dec 08 | Nov 09 | Dec 08 | Nov 09 |
| Western Highlands | Tabibuga |  |  | May 09 | Jun 10 |  |  |
| Sandaun | Yapsie(i) |  |  | Aug 09 | Aug 10 | Aug 09 | Aug 10 |
