# Supplementary material for: Changes in malaria burden and transmission in sentinel sites after the roll-out of long-lasting insecticidal nets in Papua New Guinea
Source: Parasit Vectors. 2016 Jun 14;9:340. doi: 10.1186/s13071-016-1635-x (PMC4908799; doi:10.1186/s13071-016-1635-x)
Supplement: Additional file 3: Table S3. — Morbidity indicators in two sentinel health facilities before and after LLIN distribution. (DOCX 15 kb) [file 13071_2016_1635_MOESM3_ESM.docx]

**Additional file 3: Table S3** Morbidity indicators in two sentinel health facilities before and after LLIN distribution

| **Site** | **Pre-LLIN** | | **Post-LLIN** | |  |  |
| --- | --- | --- | --- | --- | --- | --- |
|  | ***n*** | **% (95 % CI)** | ***n*** | **% (95 % CI)** | **Adj. OR (95 % CI)** | ***P*-value** |
| **Anaemia** |  |  |  |  |  |  |
| Mumeng | 463 | 52.9 (48.3, 57.5) | 216 | 40.7 (34.1, 47.6) | 0.6 (0.5, 0.9) | 0.015 |
| Sausi | 453 | 63.6 (59.0, 68.0) | 128 | 61.7 (52.7, 70.2) | 1.1 (0.7, 1.8) | 0.586 |
| Overall | 916 | 58.2 (54.9, 61.4) | 344 | 48.6 (43.2, 54.0) | 0.8 (0.6, 1.0) | 0.058 |
| **Severe anaemia** |  |  |  |  |  |  |
| Mumeng | 463 | 8.0 (5.7, 10.9) | 216 | 4.2 (1.9, 7.8) | 0.5 (0.2, 1.1) | 0.085 |
| Sausi | 453 | 9.3 (6.8, 12.3) | 128 | 6.3 (2.7, 11.9) | 0.8 (0.4, 1.8) | 0.591 |
| Overall | 916 | 8.6 (6.9, 10.6) | 344 | 4.9 (2.9, 7.8) | 0.6 (0.4, 1.1) | 0.091 |
| **Splenomegaly** |  |  |  |  |  |  |
| Mumeng | 178 | 24.2 (18.1, 31.1) | 62 | 9.7 (3.6, 19.9) | 0.6 (0.4, 1.1) | 0.016 |
| Sausi | 166 | 50.6 (42.8, 58.4) | 31 | 19.4 (7.5, 37.5) | 0.2 (0.1, 0.6) | 0.003 |
| Overall | 344 | 36.9 (31.8, 42.3) | 93 | 12.9 (6.9, 21.5) | 0.3 (0.1, 0.5) | <0.001 |
