# Supplementary material for: Changes in malaria burden and transmission in sentinel sites after the roll-out of long-lasting insecticidal nets in Papua New Guinea
Source: Parasit Vectors. 2016 Jun 14;9:340. doi: 10.1186/s13071-016-1635-x (PMC4908799; doi:10.1186/s13071-016-1635-x)
Supplement: Additional file 4: Table S4. — Morbidity indicators in the general population before and after LLIN distribution. (DOCX 19 kb) [file 13071_2016_1635_MOESM4_ESM.docx]

**Additional file 4: Table S4** Morbidity indicators in the general population before and after LLIN distribution

|  | **Pre-LLIN** | | **Post-LLIN** | |  |  |
| --- | --- | --- | --- | --- | --- | --- |
|  | ***n*** | **% (95 % CI)** | ***n*** | **% (95 % CI)** | **Adj. OR (95 % CI)** | ***P*-value** |
| **Fever > 37.5 °C** |  |  |  |  |  |  |
| Finschhafen | 441 | 0.9 (0.2, 2.3) | 412 | 1.9 (0.8, 3.8) | 2.2 (0.6, 7.3) | 0.213 |
| Mumeng | 284 | 4.2 (2.2, 7.3) | 452 | 2.0 (0.9, 3.7) | 0.3 (0.1, 0.9) | **0.022** |
| Sausi | 326 | 2.1 (0.9, 4.4) | 411 | 2.4 (1.2, 4.4) | 1.2 (0.4, 3.2) | 0.732 |
| Tabibuga | 309 | 2.6 (1.1, 5.0) | 329 | 0.9 (0.2, 2.6) | 0.3 (0.1, 1.2) | 0.087 |
| Overall | 1,360 | 2.3 (1.6, 3.2) | 1,604 | 1.9 (1.3, 2.7) | 0.7 (0.4, 1.2) | 0.208 |
| **Reported fever** |  |  |  |  |  |  |
| Finschhafen | 445 | 18.4 (14.9, 22.3) | 416 | 11.8 (8.8, 15.3) | 0.6 (0.4, 0.9) | **0.008** |
| Mumeng | 287 | 13.6 (9.8, 18.1) | 450 | 6.2 (4.2, 8.9) | 0.4 (0.2, 0.7) | **0.001** |
| Sausi | 327 | 7.6 (5, 11.1) | 411 | 2.2 (1, 4.1) | 0.3 (0.1, 0.6) | **0.001** |
| Tabibuga | 305 | 16.1 (12.1, 20.7) | 328 | 12.5 (9.1, 16.6) | 0.8 (0.5, 1.2) | 0.266 |
| Overall | 1,364 | 14.3 (12.5, 16.3) | 1,605 | 7.9 (6.6, 9.3) | 0.5 (0.4, 0.7) | **< 0.001** |
| **Anaemia** |  |  |  |  |  |  |
| Finschhafen | 446 | 76.7 (72.5, 80.5) | 415 | 58.8 (53.9, 63.6) | 0.4 (0.3, 0.6) | **< 0.001** |
| Mumeng | 171 | 65.5 (57.9, 72.6) | 453 | 38.6 (34.1, 43.3) | 0.2 (0.2, 0.4) | **< 0.001** |
| Sausi | 320 | 67.2 (61.7, 72.3) | 410 | 66.1 (61.3, 70.7) | 0.9 (0.7, 1.3) | 0.728 |
| Tabibuga | 308 | 58.8 (53, 64.3) | 325 | 34.8 (29.6, 40.2) | 0.3 (0.2, 0.5) | **< 0.001** |
| Overall | 1,245 | 68.3 (65.6, 70.9) | 1,603 | 50.1 (47.6, 52.6) | 0.4 (0.4, 0.5) | **< 0.001** |
| **Severe anaemia** |  |  |  |  |  |  |
| Finschhafen | 446 | 5.6 (3.7, 8.2) | 415 | 2.4 (1.2, 4.4) | 0.4 (0.2, 1.0) | **0.037** |
| Mumeng | 171 | 5.3 (2.4, 9.8) | 453 | 2.6 (1.4, 4.6) | 0.4 (0.2, 1.1) | 0.067 |
| Sausi | 320 | 3.8 (2, 6.5) | 410 | 2.7 (1.3, 4.7) | 0.7 (0.3, 1.7) | 0.429 |
| Tabibuga | 308 | 4.9 (2.8, 7.9) | 325 | 1.8 (0.7, 4) | 0.4 (0.1, 0.9) | **0.035** |
| Overall | 1,245 | 4.9 (3.8, 6.2) | 1,603 | 2.4 (1.7, 3.3) | 0.5 (0.3, 0.7) | **< 0.001** |
